# Clinical Imaging of the Penumbra in Ischemic Stroke: From the Concept to the Era of Mechanical Thrombectomy

**DOI:** 10.3389/fcvm.2022.861913

**Published:** 2022-03-09

**Authors:** Lucie Chalet, Timothé Boutelier, Thomas Christen, Dorian Raguenes, Justine Debatisse, Omer Faruk Eker, Guillaume Becker, Norbert Nighoghossian, Tae-Hee Cho, Emmanuelle Canet-Soulas, Laura Mechtouff

**Affiliations:** ^1^Univ Lyon, CarMeN Laboratory, INSERM, INRA, INSA Lyon, Université Claude Bernard Lyon 1, Lyon, France; ^2^Olea Medical, La Ciotat, France; ^3^Grenoble Institut Neurosciences, INSERM, U1216, Univ. Grenoble Alpes, Grenoble, France; ^4^CREATIS, CNRS UMR-5220, INSERM U1206, Université Lyon 1, Villeurbanne, France; ^5^Neuroradiology Department, Hospices Civils of Lyon, Lyon, France; ^6^Stroke Department, Hospices Civils of Lyon, Lyon, France

**Keywords:** cerebral metabolic rate of oxygen, MRI, PET, ischemic thresholds, penumbra, thrombolysis, thrombectomy

## Abstract

The ischemic penumbra is defined as the severely hypoperfused, functionally impaired, at-risk but not yet infarcted tissue that will be progressively recruited into the infarct core. Early reperfusion aims to save the ischemic penumbra by preventing infarct core expansion and is the mainstay of acute ischemic stroke therapy. Intravenous thrombolysis and mechanical thrombectomy for selected patients with large vessel occlusion has been shown to improve functional outcome. Given the varying speed of infarct core progression among individuals, a therapeutic window tailored to each patient has recently been proposed. Recent studies have demonstrated that reperfusion therapies are beneficial in patients with a persistent ischemic penumbra, beyond conventional time windows. As a result, mapping the penumbra has become crucial in emergency settings for guiding personalized therapy. The penumbra was first characterized as an area with a reduced cerebral blood flow, increased oxygen extraction fraction and preserved cerebral metabolic rate of oxygen using positron emission tomography (PET) with radiolabeled O_2_. Because this imaging method is not feasible in an acute clinical setting, the magnetic resonance imaging (MRI) mismatch between perfusion-weighted imaging and diffusion-weighted imaging, as well as computed tomography perfusion have been proposed as surrogate markers to identify the penumbra in acute ischemic stroke patients. Transversal studies comparing PET and MRI or using longitudinal assessment of a limited sample of patients have been used to define perfusion thresholds. However, in the era of mechanical thrombectomy, these thresholds are debatable. Using various MRI methods, the original penumbra definition has recently gained a significant interest. The aim of this review is to provide an overview of the evolution of the ischemic penumbra imaging methods, including their respective strengths and limitations, as well as to map the current intellectual structure of the field using bibliometric analysis and explore future directions.

## 1. Introduction

Stroke is the second most common cause of death and a leading cause of disability in occidental countries, and the 20- to 30-year projection forecasts around 20–30% increase of its burden (1, 2).

In case of acute ischemic stroke (AIS), occlusion of a cerebral blood vessel causes a variable decrease of blood flow in the downstream parenchyma. Three major zones have been identified: (i) the irreversibly damaged ischemic core; (ii) the ischemic penumbra, defined as the severely hypoperfused, electrically silent, at risk brain tissue; and (iii) the oligemia, a mildly hypoperfused area with preserved neuronal function (3, 4). As time elapses, the core progresses within the ischemic penumbra, but this area can be salvaged if perfusion is restored (5).

Therefore, the primary target of AIS treatment is to restore brain perfusion as soon as possible in order to preserve the ischemic penumbra. Reperfusion therapies, such as intravenous (IV) thrombolysis with recombinant tissue plasminogen activator (rt-PA) and mechanical thrombectomy (MT) for selected patients with large vessel occlusion (LVO), have been shown to improve functional outcome within a time window of 4.5 and 6 h, respectively (6–8).

Strict time windows, however, have been questioned as the infarct core progresses at different rates across individuals. The primary pathophysiological variable distinguishing “fast progressors” from “slow progressors” is the collateral status, and particularly the functionality of the leptomeningeal anastomoses (9). Therefore, a growing paradigm has been the shift from a “time-based” to a “tissue-based” approach, thus using a therapeutic window tailored to each patient's unique pathophysiology. Recent clinical trials that included penumbral imaging in their eligibility criteria demonstrated the benefit of reperfusion therapy beyond conventional time windows of up to 9 h for IV thrombolysis and up to 24 h for MT (10). Mapping the ischemic penumbra has therefore become critical when managing AIS patients in order to identify those who are the most amenable to reperfusion strategies beyond conventional time windows (11, 12).

The aim of this review is to provide an overview of the ischemic penumbra imaging methods from positron emission tomography (PET) imaging, the historical one, to multiparametric magnetic resonance imaging (MRI) and computed tomography (CT) that can be used as operational surrogate in AIS to guide therapeutic decisions. Using co-citation analysis, a bibliometric analysis of the field was carried out to observe the emergence of these methods. Additionally, the bibliographic coupling focusing on the MT era sketches the current state of research in order to position future contributions to the field.

## 2. [^15^O]-PET: The Gold Standard of Ischemic Penumbra Imaging

Initially, the ischemic penumbra was identified in baboons as severely hypoperfused and electrically silent tissue but without massive release of extracellular potassium. This definition relies on the measured electrical response as well as pH and potassium variation throughout the occlusion time (13, 14). [^15^O]-PET imaging on baboons validated the existence of the ischemic penumbra with metabolic parameters (3, 15). The transition from electrical activity to imaging parameters established a first shift of paradigm in the field (16).

**Figure 1:**
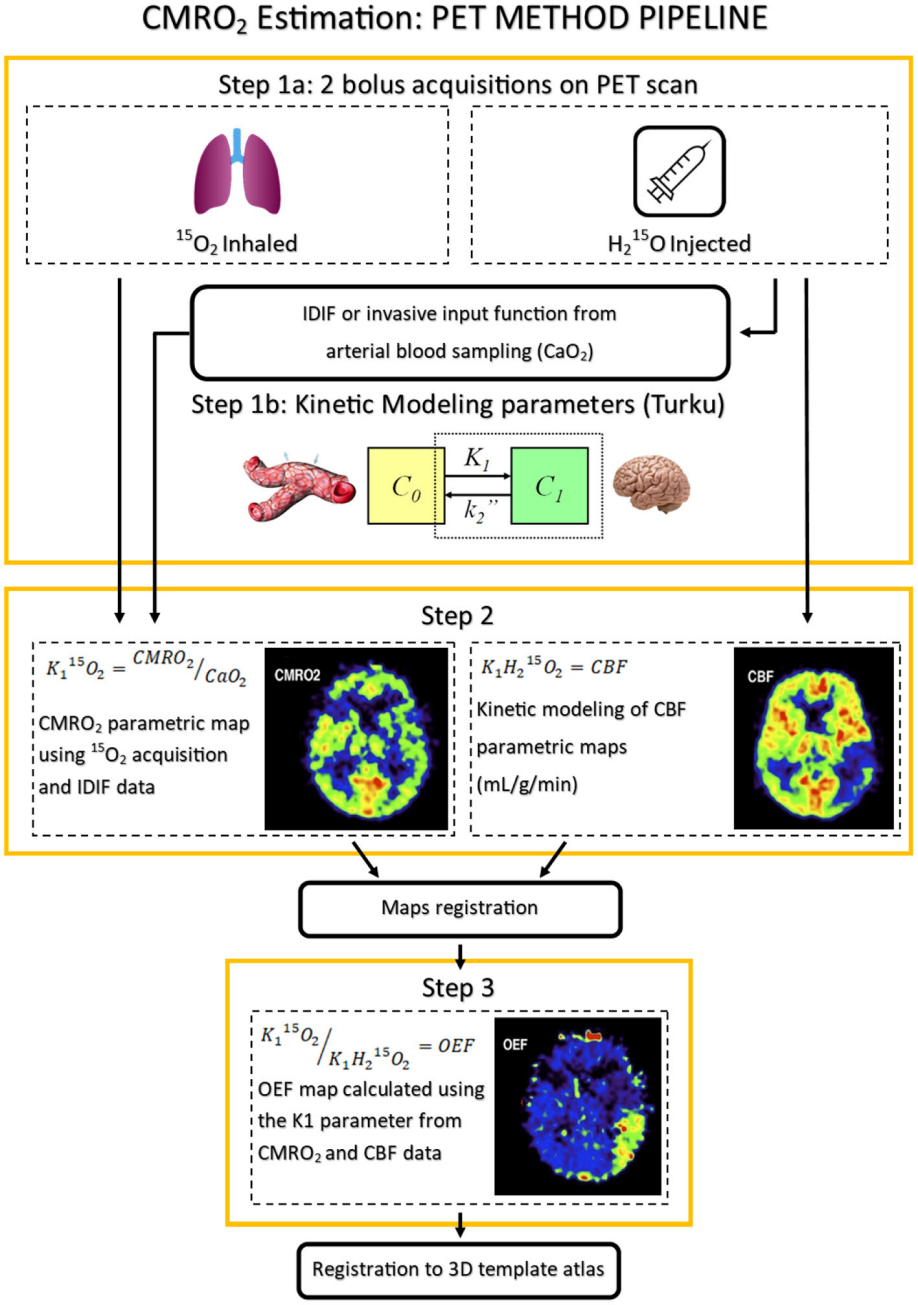
Oxygen metabolism [^15^O]-Positron Emission Tomography (PET) imaging pipeline. PET images published in JNM. Heiss WD. Radionuclide imaging in ischemic stroke. *J Nucl Med*. (2014) 55:1831–41. © SNMMI. CMRO_2_, cerebral metabolic rate of oxygen; IDIF, image-derived input function; CaO_2_, arterial blood oxygen content; VOI, volume of interest; CBF, cerebral blood flow; OEF, oxygen extraction fraction; K_1_, tracer delivery rate.

The existence of the ischemic penumbra in humans was demonstrated using PET imaging 40 years ago and confirmed the data obtained in baboons (17). The mapping of cerebral blood flow (CBF) and O_2_ metabolism biomarkers such as the oxygen extraction fraction (OEF) and the cerebral metabolic rate of oxygen (CMRO_2_) is obtained through a series of PET acquisitions following inhalation and injection of molecules labeled with^15^O radioactive tracer. The steady state method was first implemented, and consists in maintaining the tracer's concentration in the blood to a constant level throughout the acquisition period. This method was replaced by a bolus injection and inhalation method that drastically reduced the radioactive doses required for image acquisition (18). Two acquisitions are required in the imaging pipeline to obtain the CMRO_2_ parameter map (19). The injection of a labeled water ([^15^O]H_2_O) bolus provides CBF mapping. The time activity curve following the injection enables the numerical calculation of the image-derived arterial input function (IDIF) and may prevent invasive procedures to obtain the arterial blood curves (20). The second bolus acquisition is performed with an inhaled [^15^O]O_2_ tracer. Oxygen extraction from capillaries to cerebral tissues is modeled as a single-tissue compartment kinetic model providing CMRO_2_ mapping (21). These acquisitions are then combined to compute the OEF mapping as described mathematically by Kudomi et al. (20). The post-processing pipeline for metabolic penumbra imaging with [^15^O]-PET is described in Figure 1 (22).

The relevance of the cerebral oxygen metabolism in predicting tissue outcome in ischemic stroke patients was demonstrated using [^15^O]-PET imaging (23). CBF distinguished penumbra (CBF 8-20 mL/100g/min) from ischemic core (CBF <8 mL/100g/min) and oligemia (CBF 20-50 mL/100g/min) (24, 25). Although the OEF was identified as a key factor in evaluating the transition from ischemic to infarcted tissues (26), CMRO_2_ was found to be the most accurate predictive parameter (27). Infarcted regions had a significant drop of CMRO_2_ below the threshold of 1.4 mL/100g/min whereas viable tissues were characterized by a maintained oxygen metabolism level above that threshold. The ischemic penumbra PET imaging pattern, initially called “misery perfusion”, is therefore characterized as a hypoperfused region (CBF 8–20 mL/100g/min) with increased OEF and relatively preserved CMRO_2_ (CMRO_2_ ≥ 1.4 mL/100g/min) (28). These thresholds were established through longitudinal studies in AIS patients prior to any reperfusion therapy. Few data are available in the setting of IV thrombolysis (29).

[^15^O]-PET has been fundamental in demonstrating the existence of the ischemic penumbra in humans, thus driving the development of reperfusion therapies. However, it is impractical in the emergency setting and exposes patients to high doses of radioactivity (18).

Notably, two other PET radiotracers have also been proposed as markers of neuronal integrity: (i) 11C-flumazenil (FMZ) a ligand selective for the central benzodiazepine receptor, whose binding is reduced in irreversible tissue damage (30, 31); (ii) (18)F-misonidazole (FMISO) which is trapped within hypoxic cells but is not specific to the penumbra (32). Although they are both straightforward PET radiotracers compared to ^15^O, their use is not extended to the AIS emergency setting.

Alternatively, single photon emission computed tomography (SPECT) using 99mTc-HMPAO (99m-Technetium hexamethylpropyleneamineoxime) or ECD (99mTc-ethyl-cysteinate-dimer) also provides data on perfusion in brain tissue. Although relative thresholds of CBF reduction have been proposed, the combination of SPECT and diffusion-weighted MRI seems more accurate to distinguish the core from the penumbra (33–36). However, SPECT can not be used in clinical emergency setting.

## 3. Evolution of Penumbra Imaging in Clinical Emergency Settings

Reperfusion therapies, such as IV thrombolysis with recombinant tissue plasminogen activator (rt-PA) and mechanical thrombectomy (MT) for selected patients with large vessel occlusion (LVO), have been shown to improve clinical outcome in AIS patients. While penumbra imaging is not formally required for patient's eligibility in early time windows, it has been proposed for the selection of patients who are the most amenable to reperfusion strategies in later time windows. Operational penumbra imaging methods, that are feasible in emergency setting, are required for this purpose. In this context, CT and MRI modalities have been introduced to estimate the penumbra in clinical routine (37, 38).

**Figure 2:**
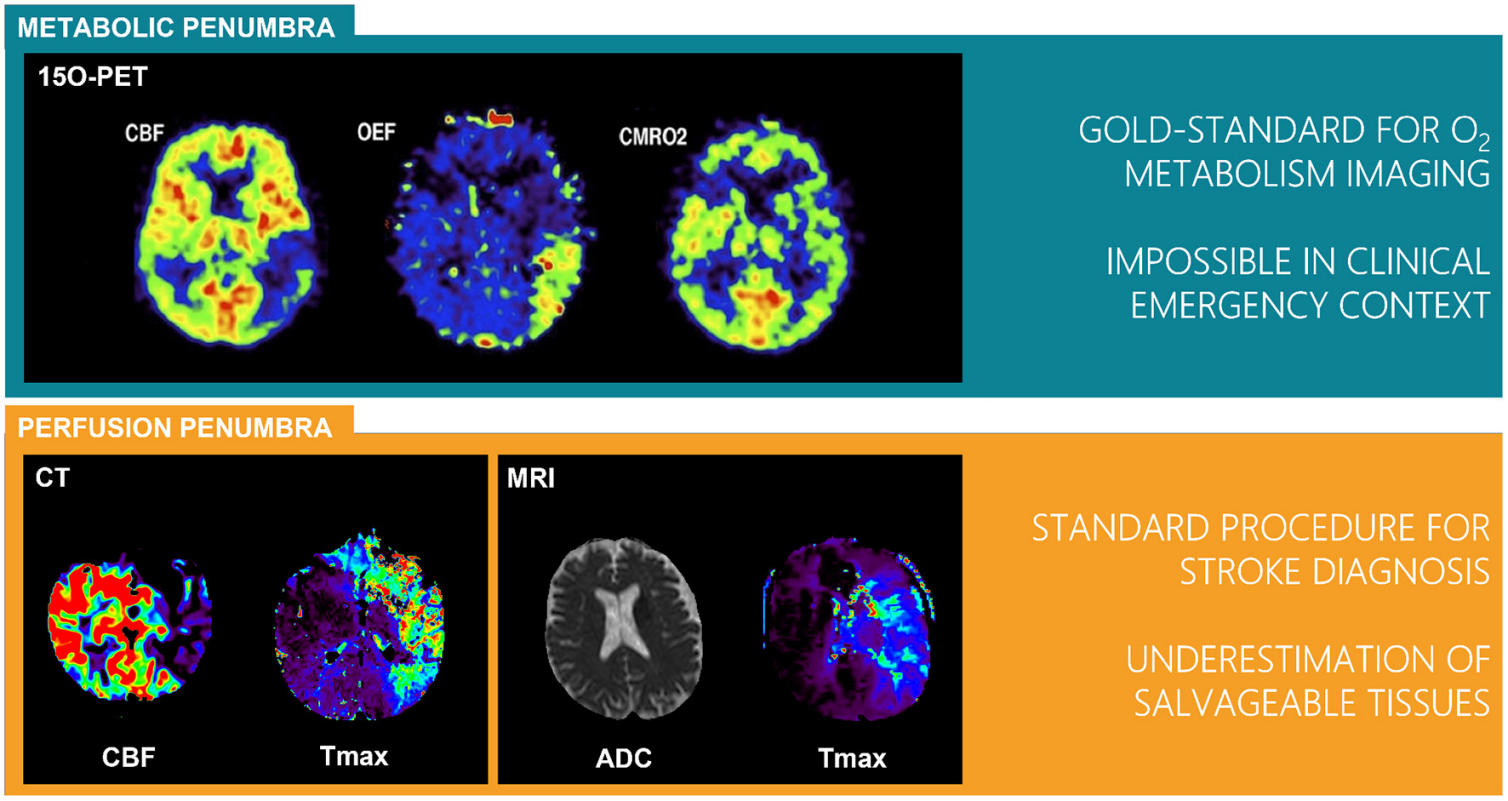
Definitions of perfusion and metabolic penumbra from the gold standard [^15^O]-Positron Emission Tomography (PET) to the widely distributed Computed Tomography (CT) and Magnetic Resonance Imaging (MRI) perfusion imaging. PET images published in JNM. Heiss WD. Radionuclide imaging in ischemic stroke. *J Nucl Med*. (2014) 55:1831–41. SNMMI.; © CT and MRI images from Olea Medical. CBF, cerebral blood flow; CMRO_2_, cerebral metabolic rate of oxygen; OEF, oxygen extraction fraction; ADC, apparent diffusion coefficient; Tmax, time to maximum of residual function.

Hemodynamic parameters maps can be computed using CT or MR perfusion imaging. Perfusion data processing requires the manual or automatic selection of an arterial input function, which is used for the deconvolution of each voxel's concentration time curve. Deconvolution can be done using a variety of techniques, ranging from the classical singular value decomposition (SVD)-based algorithms (39) to more advanced Bayesian approaches (40). Perfusion parameters such as the cerebral blood flow (CBF), cerebral blood volume (CBV), mean transit time (MTT), time to peak (TTP), time to maximum (Tmax), or the arterial delay can be used to distinguish tissue with a perfusion deficit from the infarct core and the normal tissue. Furthermore, the apparent diffusion coefficient (ADC) parameter derived from diffusion weighted MRI (DWI) is an excellent surrogate for detecting the infarct core (41, 42). A new definition based on the mismatch between infarct core and the tissue with a perfusion deficit provides the perfusion penumbra as opposed to the originally defined metabolic penumbra obtained with [^15^O]-PET. A parallel between these two penumbra can be found on Figure 2 (22).

To differentiate the penumbra from the oligemia, perfusion thresholds were initially defined using relative CBF and CBV parameters opposed to the contralateral control region (CCR) (38, 43). With the development of deconvolution methods, a longitudinal study conducted in a small sample of patients treated with IV thrombolysis has shown that a Tmax > 6s provides an accurate estimate of critically hypoperfused tissue in MRI (44); the same threshold was later approved for CT. This threshold is commonly used to obtain the perfusion penumbra by subtracting the lesion core. This definition has been used as a criteria for selecting patients in clinical trials using the RAPID software (iSchemaView Inc., Menlo Park, CA) for image processing (11).

**Figure 3:**
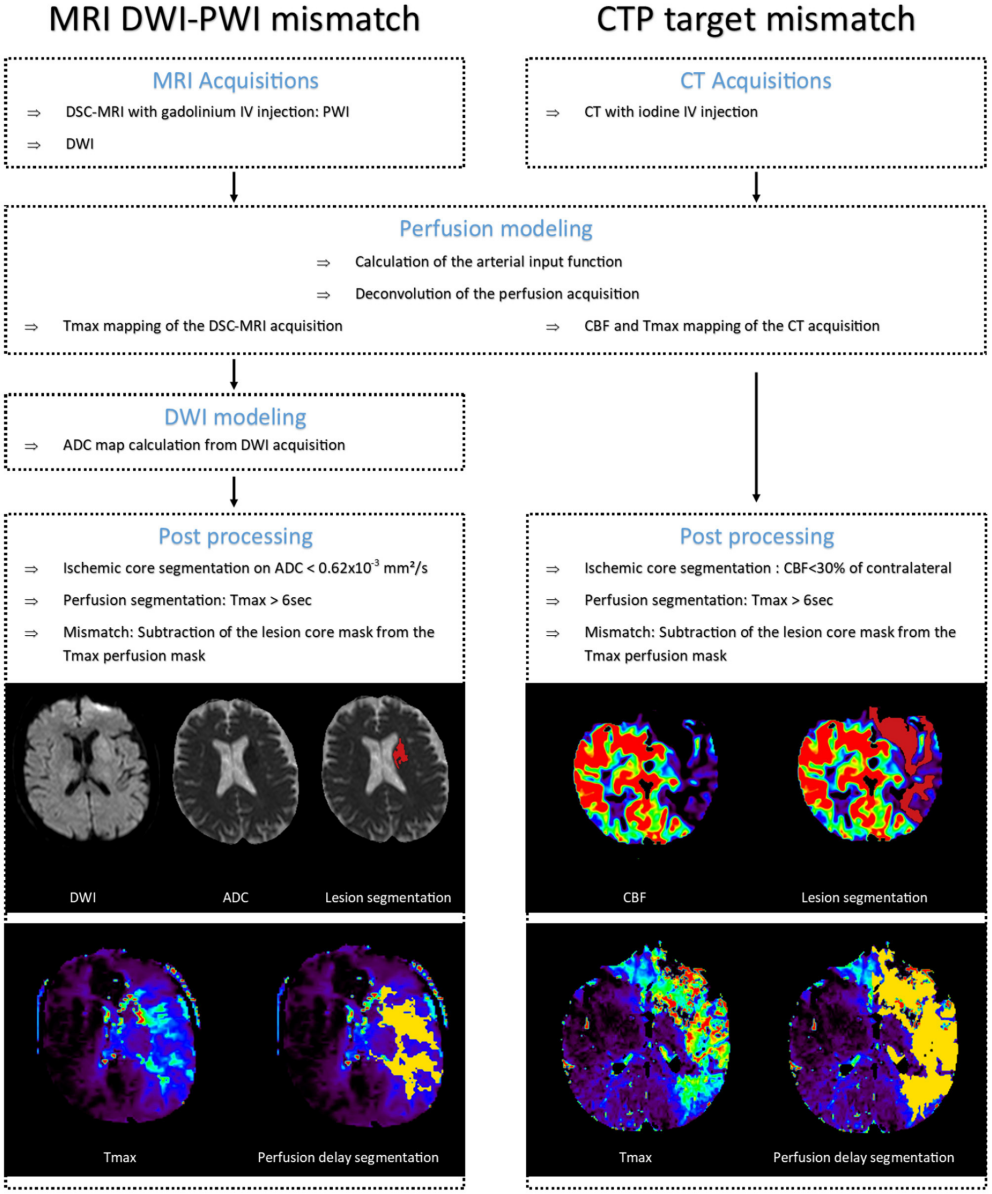
CT and MRI pipelines. In dark red: segmented infarct core. In yellow: segmented perfusion delay on Tmax. Images from Olea Medical. MRI, magnetic resonance imaging; DWI, diffusion weighted imaging; PWI, perfusion weighted imaging; CTP, computed tomography perfusion; DSC, dynamic susceptibility contrast; IV, intravenous bolus; CBF, cerebral blood flow; Tmax, time to maximum of residual function; ADC, apparent diffusion coefficient.

The lesion core definition, on the other hand, differs between the two imaging modalities. In MRI, the DWI-derived ADC parameter distinguishes the core with a threshold of ADC <0.62 × 10^−3^mm^2^\s (45). However, methods to identify the lesion core on ADC maps may vary and semi-automated as well as manual methods are still favored in clinical settings. In contrast, with CT imaging, the CBF parameter derived from perfusion acquisition defines the core in opposition to the CCR (CBF <30% of CCR) (46). Figure 3 summarizes the pipeline of both modalities.

CT and MRI have been used in clinical trials to select patients with persistent penumbra in order to assess the benefit of reperfusion therapies in later time windows. These trials have demonstrated that reperfusion therapies benefit these selected patients for up to 9 h for intravenous (IV) thrombolysis (10) and up to 16–24 h for MT (11, 12). The shift from a “time-based” to a “tissue-based” approach has significantly improved stroke management by extending therapeutic windows in patients with persistent penumbra, i.e. those with a good collateral status (47, 48). CT and MRI are therefore used in daily practice to guide therapy in patients managed beyond the conventional time window. While clinical trials used RAPID or Olea Sphere with above-mentioned thresholds for image processing, other post-processing software (Philips, Siemens, Vitrea) have been developed with alternative perfusion imaging thresholds adapted to the deconvolution method. However comparative studies have shown significant differences in their predictive accuracy of final infarct volume in the setting of MT (49, 50).

**Figure 4:**
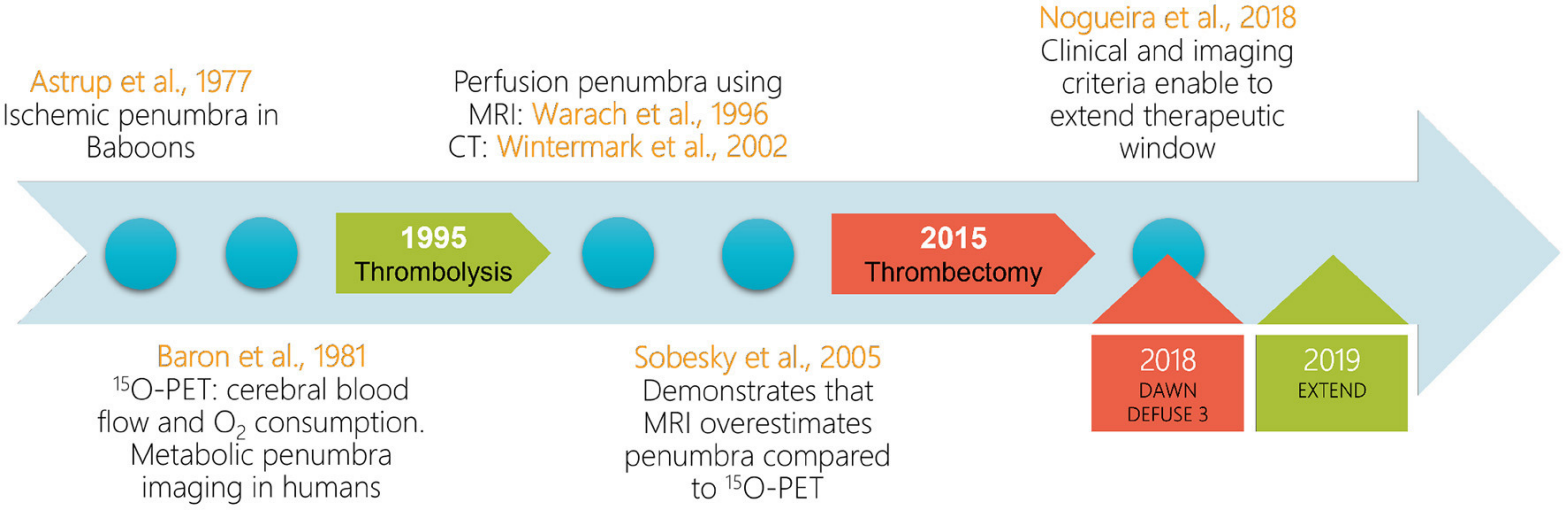
Summarized development timeline of the penumbra imaging field. Circles: the major contributions to the acute ischemic stroke research field. Arrows: the clinical validation years for reperfusion therapies. Rectangles: the major clinical trials using advanced imaging to extend therapeutic windows. Red and green code for mechanical thrombectomy and intravenous thrombolysis clinical trials, respectively. PET, positron emission tomography; MRI, magnetic resonance imaging; CT, computed tomography.

This historical association between imaging and AIS management was followed by a fast evolution of therapeutic and imaging methods in the field of AIS care. Figure 4 illustrates the timeline with the major evolutions and key-factors.

The scientific literature review of this field is a very challenging task as there are over 1000 articles on penumbra imaging in scientific databases, with about 50% of these articles published since 2015, in the era of MT. Therefore, in terms of data quantity, bibliometric analysis was a relevant approach to provide an overview of the field and an insight into the knowledge of the thrombectomy era. The bibliometric analysis was carried out in accordance with the methodology recommendations of Donthu et al. (51) and the material and methods used for this analysis can be found in Supplementary Material.

**Figure 5:**
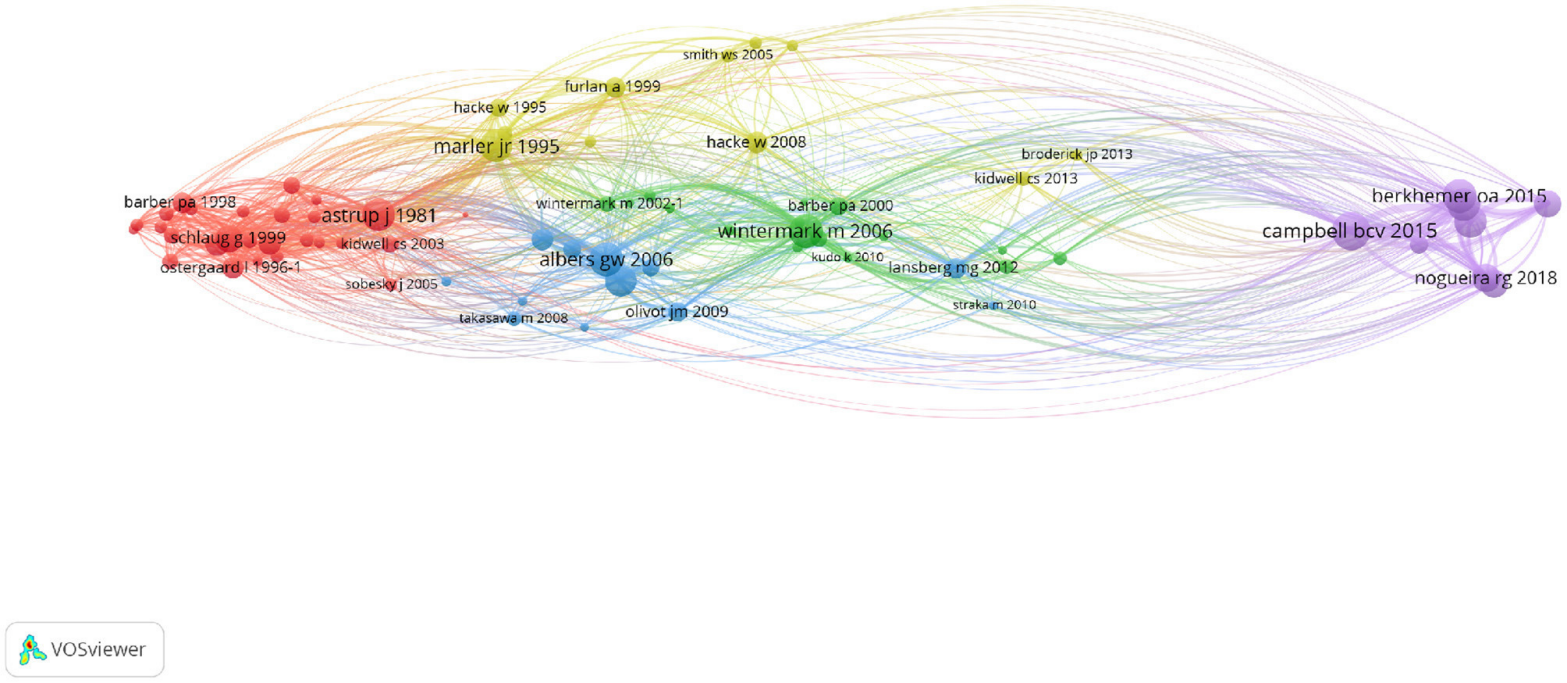
Co-citation network providing an overview of the acute ischemic stroke research field evolutions through its most contributing publications: Red cluster [1981–2005]: MRI diffusion-perfusion mismatch; Green cluster [2000–2013]: perfusion CT; Blue cluster [2003–2012]: Eligibility for thrombolysis; Yellow cluster [1995–2013]: Thrombolysis combined to endovascular therapies; Purple cluster [2015–2018]: Mechanical thrombectomy. MRI, magnetic resonance imaging; CT, computed tomography.

The co-citation analysis is a bibliometric method that calculates how frequently two articles are cited together. It uncovers fundamental publications and enables a mapping of the field's foundation knowledge and evolutions. The analysis included the 75 most co-cited documents. This threshold increased the network's legibility and highlighted the most important contributions. The resulting network can be found in Figure 5 where cluster denomination is based on predominance of the topic.

The applied mapping method retraces the timeline developed in Figure 4. Thus, the query defined for data collection (described in Supplementary Material) is relevant as the fundamental knowledge of the field obtained through co-citation analysis is similar to the current understanding of the field. The resulting clusters reveal the main research topics in the field as well as their interdependence. These observations, along with the geographical position (physical location within the network) of the various network elements, provide additional interpretation material.

While the majority of the articles in the red cluster relates to MRI sequences to evaluate lesion core and perfusion delay mismatch (16/27 articles), it also includes articles about threshold and biomarkers of penumbral tissues using PET, CT, and MRI. These articles represent 10/27 documents within this cluster. This cluster also includes the first publication defining the ischemic penumbra (3). The detailed exploration of the content enables the following elaborations: this cluster contains all major articles on penumbra definition from the theory to the first clinical applications through new thresholds definitions and comparison to [^15^O]-PET imaging. Several studies have compared MRI to [^15^O]-PET back-to-back in ischemic stroke patients and demonstrated that MRI overestimated the penumbra (52, 53). Two major limitations for MRI definition of the penumbra have been identified: (i) ADC reduction is considered to be a reflection of the ischemic core but a part of the ADC lesion may reverse (54); (ii) Distinguishing perfusion deficit from oligemia is challenging and several thresholds have been proposed, as previously demonstrated.

All of the articles in the green cluster are focused on CT imaging. Perfusion CT and associated thresholds are covered in 10/16 publications. 3 articles elaborate on other CT imaging modalities [Dynamic CT perfusion (46) and CT angiography (55)]. 2 publications relate to the ASPECTS clinical trial (56, 57). Finally, one publication compares CT perfusion method to MRI diffusion-perfusion mismatch (58). This cluster is centered on CT imaging methods in AIS management from the universally accepted scoring system to specific methods to evaluate ischemic tissue perfusion. Because of its rapid feasibility and widespread availability, CT has been the first-line imaging procedure in a majority of countries worldwide. In the 16 major clinical trials conducted since 2012, while 50% based their eligibility criteria on either CT or MR imaging, 43.75% were focused only on CT and 6.25% only on MRI (59, 60). This reflects the use of CT as the primary imaging modality for AIS in most stroke units in daily practice due to high availability, lack of contraindications, and reduced scan-duration. However, MRI is highly feasible in AIS setting, offers additional information regarding the tissue state and prevents from radiation exposure without introducing longer delay compared with the CT-selected patients and may improve outcome despite the potential delays in workflow time metrics (61–65).

The blue cluster contains a majority of articles related to thrombolytic therapies (7/12). Within this subcluster, 5 articles are related to clinical trials that aimed to extend therapeutic time windows [DIAS (66), DIAS-2 (67) and EPITHET (68)] or identifying MRI findings of patients who are likely to benefit from reperfusion therapies in later time window [DEFUSE (69, 70)]. In addition, 3 articles are focused on the post-processing MRI sequences: (i) The reference article on SVD deconvolution for MR perfusion (39); (ii) A retrospective analysis of the EPITHET clinical trial aiming to define a standardized MRI procedure for perfusion-diffusion mismatch (71); (iii) The RAPID solution for automating the task (72). Notably, two publications aimed to compare MRI and PET imaging of the ischemic penumbra (73, 74). Thus, this cluster introduces reperfusion therapy along with clinical trials aiming to increase therapeutic windows as well as imaging methods for selecting patients.

The yellow cluster relates to reperfusion therapies and is almost evenly split between IV thrombolysis (6/11) and MT (4/11) as well as combined methods. Three publications are related to IV thrombolysis clinical trials [PROACT II (75), ECASS II (76), ATLANTIS, and NINDS (77)]. The 4 articles related to MT refer to clinical trials for clot retriever devices (78–80) and imaging eligibility criteria (81). This cluster demonstrates the connection between the two treatments, frequently combined in clinical settings (82).

The last cluster, in purple, is centered on thrombectomy with a majority of publications referring to the major clinical trials demonstrating the benefit of MT and extending the therapeutic window with two subclusters in 2015(5/9) and 2018(2/9). In 2015, MR CLEAN was the first to demonstrate that MT outperformed IV thrombolysis alone in patients within 6 h of last known normal with LVO (83). Five other clinical trials [EXTEND IA (84), SWIFT PRIME (85), REVASCAT (86), ESCAPE (87), THRACE (88)] published in 2015 and 2016 and conducted in selected patients up to 12 h from stroke onset have confirmed these results. Among these clinical trials, 4 (EXTEND IA, SWIFT PRIME, REVASCAT, ESCAPE) included advanced imaging to select patients with limited core or good collateral circulation. In 2016, a meta-analysis of individual patient data enrolled in the 5 first clinical trials showed that MT reduced disability at 3 months with a number needed to treat 5 to prevent 1 patient from experiencing disability (8). Therefore, AHA guidelines, also found in this cluster (89), recommended to treat AIS patients with LVO and ASPECTS^1^≥6, although the role of additional imaging-based eligibility criteria is not well-established (90). In 2018, DEFUSE 3 and DAWN extended the therapeutic window to 16 and 24 h from time last known well, respectively, in highly selected patients with persistent penumbra (11, 12). DEFUSE 3 defined the penumbra with advanced imaging, CT or MRI, whereas DAWN defined the penumbra as a mismatch between the severity of the clinical deficit and the infarct volume. This last cluster highlights the key role of penumbra imaging in recent clinical trials that improved AIS management. Moreover, additional elaborations are possible knowing that 50% of the articles in this field were published since 2015, it demonstrates the major impact of these clinical trials on further research.

The detailed analysis of each cluster reveals a general trend that is shared by all clusters: the interdependence of imaging assessment, clinical trials and therapeutic solutions and windows. The recurrent presence of PET comparison with other imaging methods, on the other hand, is representative of the ongoing reassessment of relevance for clinical alternatives to penumbra imaging. The mapping obtained with the co-citation analysis is representative of the intellectual structure of the field. Furthermore, this analysis demonstrates the major publications and fundamental knowledge that serve as foundation for research in the thrombectomy era.

## 4. Penumbra Imaging in the Thrombectomy Era

The impact of MT and associated clinical trials on research for AIS management was compelling as in the course of 6 years over 500 articles were published on the topic (query described in Supplementary Material). The bibliographic coupling method was chosen in order to review this large corpus of articles.

**Figure 6:**
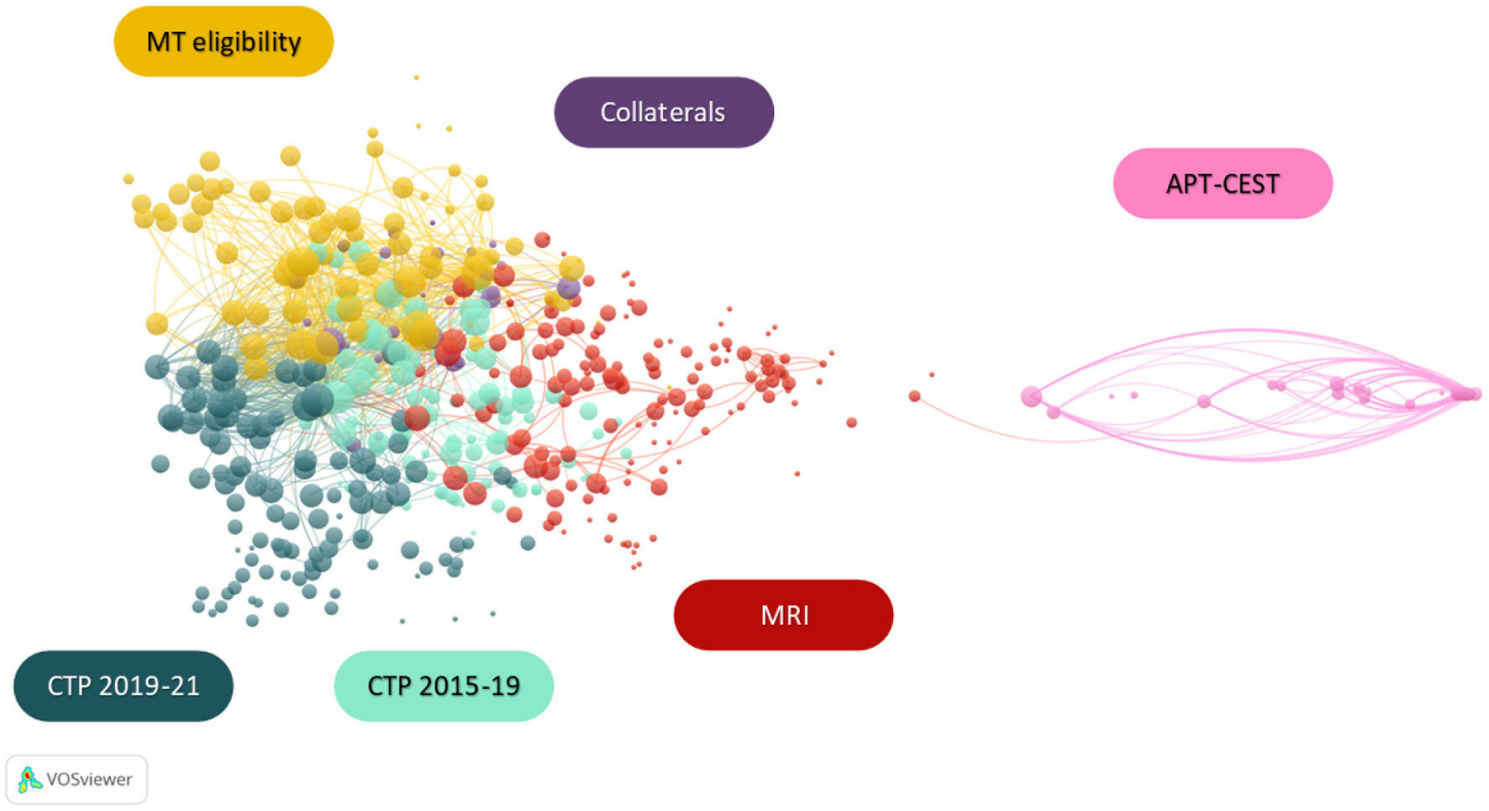
Bibliographic coupling network in the era of mechanical thrombectomy [2015–2021] with labeled clusters based on predominance of themes within the clusters. MRI, magnetic resonance imaging; CTP, computed tomography perfusion; MT, mechanical thrombectomy; APT, amide proton transfer; CEST, chemical exchange saturation transfer.

The bibliographic coupling is the process of associating articles based on the number of references they share (91). This analysis is based on the hypothesis that articles sharing a large number of common references have similar content. The aim for this technique is to provide an intellectual mapping of the field by identifying thematic clusters. Applied to a specific time frame, it uncovers niche themes as well as recent development of the field (51). In the current review, all articles published since 2015 that matched the query described in Supplementary Material were included in the analysis. Figure 6 presents an annotated version of the network with cluster nomination based on the predominance of a theme within the cluster. The Supplementary Material contains the original figure as well as the described calculation method.

The resulting figure is densely populated and therefore interpretation of the bibliographic coupling is a challenging task. Following guidelines described in Supplementary Material, the most connected articles of each cluster were reviewed in order to elaborate on their content.

The presence of collaterals and MT eligibility clusters shows the emergence of tissue based patient selection criteria and a more personalized medicine. It also appears in imaging method clusters where tissue characterization is more and more present throughout time.

The majority of articles in the collaterals thematic cluster explore the relation between collateral circulation and infarct core growth (92–94). Other publications use MRI and CT imaging to identify markers of collateral status (95, 96).

The MT eligibility thematic cluster contains a selection of articles assessing the relevance of computed tomography perfusion (CTP) and MRI in patient selection, either with a comparison of the two methods (59, 97), or with the expression of a need for alternative imaging methods (98). This is consistent with the publications promoting tissue-based eligibility criteria over the conventional time windows (99–101). This cluster also includes guidelines intended to personalize AIS management through advanced imaging assessment (102, 103).

The cluster's thematic predominance and time frame also highlighted a change of course in the CTP research field in 2019. A detailed analysis of these clusters revealed the evolution of CTP imaging considerations in AIS management. The first cluster ranging from 2015 to 2019 contains a majority of publications aimed at validating perfusion thresholds and determining reliable eligibility parameters from this imaging modality (104–107). It also shows the emergence of studies to evaluate core and perfusion penumbra volumes as well as their relationship to collateral circulation (32, 108). The second cluster ranging from 2019 to 2021 includes publications retrospectively analyzing CTP data from the major clinical trials concomitant with the development of MT, confirming the growing interest in imaging-based patients selection for reperfusion therapies (109–111). This time frame also shows an emergence of automated CTP imaging post-processing to provide target mismatch volume as well as infarct growth prediction (112–116).

Publications aiming to automate post-processing of imaging modalities are also largely represented in the MRI cluster with articles describing numerous methods based on machine learning (117–119). This tendency is encouraged by the need for fast identification of penumbral tissue. Additionally, this group contains a subcluster focused on the validation of MRI for MT eligible patient selection, including comparisons with PET imaging (120, 121). If the majority of the articles validating MRI are focused on PWI-DWI mismatch, however, MRI modalities and sequences providing additional parameters such as hypoperfusion with DWI-susceptibility weighted imaging (SWI) mismatch are also present in this cluster (122, 123).

These imaging modality-focused clusters are representative of the central position of imaging in AIS care. The most frequent eligibility criteria for clinical trials on reperfusion therapies are CTP target mismatch and MRI DWI-PWI mismatch. However, because they differ in defining the lesion core, the accuracy of those methods is often compared. While these two methods are widespread, other imaging modalities, not relying on perfusion imaging, have been proposed. The DWI-FLAIR (Fluid-Attenuated Inversion Recovery) mismatch is a surrogate marker of lesions within 4.5 h of symptom onset used as selection criteria in the WAKE-UP trial that assessed the benefit of IV rt-PA in patients with unknown time of onset of stroke (124). Regarding CT, multiphase CT angiography (CTA) provides information on collateral status and has been used to select patients in the ESCAPE-NA1 trial that tested the efficacy and safety of nerinetide in AIS patients treated with MT within a 12 h window (125, 126). Of interest, patients included in the latter trial (control arm) had similar outcome than patients included in DAWN and DEFUSE-3 trials despite having larger lesion volume.

The thematic predominance of each cluster in relation to their physical position within the network also provides elaboration material. While the five groups on the left represent generic concepts, the isolated cluster on the right is focused on the specific amid proton transfer modality of chemical exchange saturation transfer imaging (APT-CEST). Additionally, the MRI cluster is closer and marginally connected to the APT-CEST cluster as the latter is an imaging modality of the former. In the context of ischemic stroke, APT-CEST provides pH-weighted imaging, a marker of tissue micro-environment which variation influences hemoglobin affinity for O_2_ (127, 128). The presence of this cluster is an evidence of the growing need for imaging parameters representative of tissue activity and metabolism rather than perfusion delay markers.

## 5. Future Directions

As initially demonstrated with PET imaging, penumbral tissues are described by a relatively preserved O_2_ metabolism within a region with perfusion deficit. With the change of paradigm operating and promoting tissue based eligibility criteria for reperfusion therapies, there is a need for an operational imaging parameter to evaluate the metabolic state of the tissues. The current knowledge of the field raises legitimate concerns about other imaging modalities providing metabolic parameters.

An additional exploration of the bibliometric coupling network was carried out in order to further investigate the location of oxygen metabolism imaging methods. The method of investigation is described in Supplementary Material.

The detailed exploration of the clusters has shown that oxygen metabolism related biomarkers (OEF and CMRO_2_) imaging methods are split into 2 clusters. Three articles are contained within the MRI cluster and relate to MRI based modalities. The CTP [2019–2021] cluster contains 3 articles related to these biomarkers, however, 2/3 methods in this group are based on MRI modalities. Regarding the methods developed, 4/6 publications are based on variations of blood oxygen level dependent (BOLD) functional MRI principles (129–132). Another article provided OEF mapping through quantitative susceptibility mapping MRI (133). Lastly, dynamic CTP provided OEF and CMRO_2_ mapping based on extrapolated data from perfusion acquisition; this method remains theoretical (134).

In addition to the search for these specific biomarkers, the bibliographic coupling also contains a selection of alternative marker imaging based on hemodynamics. The MRI cluster presents 3 articles for hemodynamic assessment as a marker of tissue activity with MRI SWI (135, 136) and MRI T2^⋆^-weighted imaging (137). Two articles relate to near infrared spectroscopy for hemodynamic, side-bed, AIS patient monitoring. This imaging modality, as well as photoacoustic, do not provide whole brain imaging and are therefore not addressed in this review.

**Figure 7:**
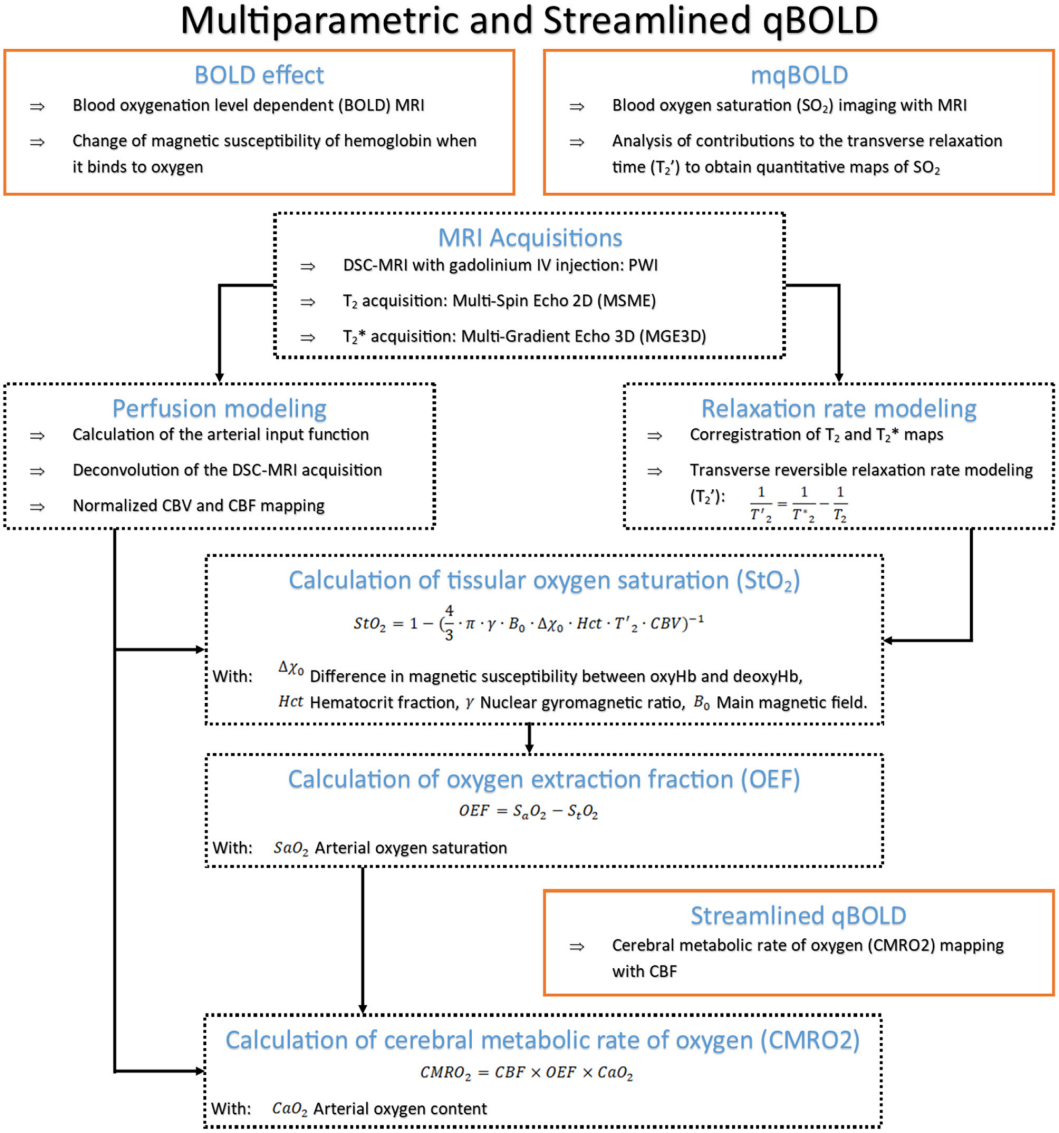
Detailed principle and pipeline for OEF mapping with the multiparametric quantitative Blood Oxygen Level Dependent (mqBOLD) method and CMRO_2_ mapping with streamlined qBOLD. MRI, magnetic resonance imaging; DSC, dynamic susceptibility contrast; IV, intravenous bolus; CBV, cerebral blood volume; CBF, cerebral blood flow.

From 2015 to 2021, articles providing metabolic parameters accounted for less than 2% of the corpus of scientific literature. Their importance within this network is minor compared to the imaging concepts that have been studied for the past two decades. The widespread distribution of these articles throughout the network indicates that the scientific community fails to reach a consensus on oxygen metabolism marker imaging, but MRI methodologies predominate. Table 1 provides an overview of these MRI methods, the majority of which are based on the quantification of the BOLD effect. An example of a multiparametric qBOLD approach is described in Figure 7.

**Table 1:** Overview of MRI metabolic penumbra imaging methods.

**MRI Method**	**Description**	**Advantages**	**Limits**	**Imaging parameters**	**References**
^17^O	Similar concept to ^15^O-PET. Two methods: - Direct method with MR spectroscopy at high field - Indirect method with proton MRI of metabolized ^17^O into [^17^OH_2_O]	- Oxygen-17 naturally occurring, chemically stable, non-radioactive isotope - MR visible and detectable directly using MR spectroscopic techniques and indirectly with proton MRI methods	- Limited availability of ^17^O marker - Low signal on clinical scanners and limited availability of high field MRI - Requires specialized coils to generate images using direct detection techniques	CMRO_2_ OEF	(138) (139)
qBOLD	Initial method using BOLD effect and from which originated mqBOLD and streamlined qBOLD	- Non-invasive - Single sequence	- Requires very high SNR - Not available in clinical settings - Multiple parameters estimation brings measurement uncertainties	Blood oxygen saturation (SO2) CBV	(140)
mqBOLD	Figure 7 provides a detailed description of the method	- Better fitting of the parameters compared to qBOLD	- Requires multiple sequences - Estimations uncertainties in white matter	SO2 CBV	(141) (142)
Streamlined qBOLD	Figure 7 provides a detailed description of the method	- Non invasive method - Enables mapping of CMRO_2_ parameter	Estimating [deoxyhemoglobin] negates the requirement for an assumed or measured haematocrit, which is required in order to estimate OEF	CBF CMRO_2_	(131)
QSM	- QSM is a postprocessing technique that quantifies local tissue magnetic properties through the solution of the field-to-source problem - Based on the magnetic susceptibility of deoxyhemoglobin in cortical veins	- Acquisition simplicity and reasonable time requirements - Easy calibration to absolute OEF	- OEF measurements extrapolated from shift of susceptibility between water and venous blood - OEF ratio between hemisphere enabled but no mapping obtained.	Quantitative susceptibility map (QSM) Spatial profiles of OEF	(133) (143)
QSM+qBOLD	This method combines the QSM and qBOLD modalities for oxygen metabolism mapping	This method is non-invasive	- A large number of acquisition is required - The mapping of the parameter relies on the clustering of voxels through the hypothesis that similar voxel signals have similar model parameter values	CMRO_2_ OEF	(132) (144)

*MRI, magnetic resonance imaging; CMRO_2_, cerebral metabolic rate of oxygen; OEF, oxygen extraction fraction; BOLD, blood oxygen level dependent; QSM, quantitative susceptibility mapping; qBOLD, quantitative BOLD; mqBOLD, multiparametric qBOLD; DSC, dynamic susceptibility contrast; PET, positron emission tomography; SNR, signal to noise ratio*.

It seems that these methods have the potential to provide the relevant markers of the ischemic penumbra. Yet, the current state of research in this field has not reached clinical validation. The methods described in Table 1 have been either validated for other pathologies or validated on ischemic stroke preclinical-models. Therefore, there are no known thresholds to qualify the metabolic penumbra in AIS clinical context with these methods. Moreover, technical issues still remain.

The original qBOLD method (140) is fast and non-invasive. However, it requires a special MR sequence usually not accessible in a clinical environment and the estimates are only valid if the signal to noise ratio (SNR) is very high. The mqBOLD approach (142) can be used with standard MR sequences but it requires the injection of a contrast agent and the co-registration of the maps is a challenging task due to the variations in resolution and potential distortions within the sequences. Moreover, the numerous parameters estimated for the calculations and the corresponding models only allows an approximated mapping of CMRO_2_ and OEF without considering tissue specificity (such as white vs. gray matter). An exciting alternative consists in the fusion of the qBOLD approach with a new MRI framework called MR fingerprinting (MRF) where complex MR sequences are directly linked to advanced numerical simulations (145). This new perspective would enable to shorten acquisition time and allow the quantification of several biomarkers simultaneously. Initial studies using MRF to quantify brain oxygenation in both humans and rodents (146, 147) have been very encouraging and the method is now under validation in preclinical AIS models and future intended contributions aim in defining thresholds for the metabolic penumbra to extend it to clinical settings.

In the present review, MRI advances are vastly discussed, but there are also developments for CTP, e.g. the calculation of penumbra and core from multiphase CTA acquisitions (148).

## 6. Conclusion

Conducting a bibliometric analysis of the field enabled to describe penumbra imaging over time and its close parallel relationship with the change of paradigm operating in AIS management. The key role of imaging was highlighted as well as the necessity for biomarkers of the cerebral tissue metabolic state in clinical emergency settings. Up to date the scientific community fails to reach a consensus on imaging and post processing modalities to meet these new requirements. MR CMRO_2_ methods and MR vascular fingerprinting for AIS models are particularly promising and deserve further exploration.

## Author Contributions

EC-S, LM, and TB proposed and initiated the review focus and scheme. LC designed, conducted, and analyzed the bibliometric study. LM supervised the manuscript drafting and clinical focus. The CMRO_2_ pipelines were initially designed by JD and EC-S and adapted by LC. The other figures were prepared by LC and overviewed by LM, TB, and EC-S. DR provided the clinical images for the figures. LC drafted the manuscript and interpreted the bibliometric data with LM, EC-S, and TB. LC, TB, TC, JD, OE, GB, NN, T-HC, EC-S, and LM reviewed the literature in their respective field of expertise and revised the manuscript critically for important intellectual content. All authors approved the final version of the manuscript submitted.

## Funding

This work was supported by ANR CYCLOPS and CMRO_2_ (ANR-15-CE17-0020 and ANR-21-CE17-0028), the RHU MARVELOUS (ANR-16-RHUS-0009) of Lyon University, under the Investissements d'Avenir program of the French National Research Agency (ANR). The Ph.D. salary of LC (Cifre, Olea Medical) is co-funded by the French Ministry of Higher Education and Research (ANRT).

## Conflict of Interest

LC, DR, and TB are employees of Olea Medical, a company developing the OleaSphere platform including pipelines for ischemic penumbra. The remaining authors declare that the research was conducted in the absence of any commercial or financial relationships that could be construed as a potential conflict of interest.

## Publisher's Note

All claims expressed in this article are solely those of the authors and do not necessarily represent those of their affiliated organizations, or those of the publisher, the editors and the reviewers. Any product that may be evaluated in this article, or claim that may be made by its manufacturer, is not guaranteed or endorsed by the publisher.

## Footnotes

^1^ASPECTS, Alberta Stroke Program Early CT Score.

## Abbreviations

ADC: apparent diffusion coefficient
AHA: american stroke association
AIS: acute ischemic stroke
APT: amide proton transfer
ASPECTS: alberta stroke program early CT score
BOLD: blood oxygen level dependent
CBF: cerebral blood flow
CBV: cerebral blood volume
CCR: contralateral control region
CEST: chemical exchange saturation transfer
CMRO_2_: cerebral metabolic rate of oxygen
CT: computed tomography
CTP: CT perfusion
DSC: dynamic susceptibility contrast
DWI: diffusion weighted imaging
FMZ: 11C-flumazenil
FMISO: (18)F-misonidazole
FLAIR: fluid-attenuated inversion recovery
IV: intravenous
LVO: large vessel occlusion
mqBOLD: multiparametric qBOLD
MRI: magnetic resonance imaging
MRF: MR fingerprinting
MT: mechanical thrombectomy
MTT: mean transit time
OEF: oxygen extraction fraction
PET: positron emission tomography
PWI: perfusion weighted imaging
qBOLD: quantitative BOLD
QSM: quantitative susceptibility mapping
rt-PA: recombinant tissue plasminogen activator
SNR: signal to noise ratio
SPECT: single photon emission computed tomography
SVD: singular value decomposition
SWI: susceptibility weighted imaging
Tmax: time to maximum of residual function
TTP: time to peak.
