# Supplementary material for: Clinical Imaging of the Penumbra in Ischemic Stroke: From the Concept to the Era of Mechanical Thrombectomy
Source: Front Cardiovasc Med. 2022 Mar 9;9:861913. doi: 10.3389/fcvm.2022.861913 (PMC8959629; doi:10.3389/fcvm.2022.861913)
Supplement: Supplementary file 1 [file Data_Sheet_1.PDF]

frontiers

Supplementary Material

1 SUPPLEMENTARY DATA

1.1 Bibliometric analysis: Material and Method

The Bibliometric analysis review type was favored over meta-analysis and systematic literature review as the scope of the study is broad. Moreover, the large dataset obtained for this analysis is too large to consider a manual review of the literature. The goal of this analysis is to present the intellectual structure and the emerging trends of the field.

1.1.1 Scope and aim of the analysis

The first step of the bibliometric analysis guideline is to define the aim and the scope of the study. For the present review, the aim is to observe the evolution of the ischemic penumbra definition with imaging methods, from its historical characterization by Astrup and al. in 1981 to the current state of research for a precise clinical definition to adjust therapeutic time windows.

1.1.2 Selecting bibliometric analysis techniques

The next step is to choose the appropriate literature analysis method. The bibliometric analysis technique most appropriate for this objective is the science mapping to examine the relationship between research constituents. In order to obtain the trends evolution overview, co-citation analysis and bibliographic coupling were carried out.

- The co-citation analysis uncovers fundamental publications and enables a mapping of the foundation knowledge in the field.
- The bibliographic coupling provides thematic clusters within time frames and is used in this review to present the current state of research.

As opposed to the method described by Donthu et al., 2021, the co-word analysis was not interpreted in this review as the obtained clustering and mapping was not representative of the field, therefore not relevant for the review. However, the detailed method bringing to this conclusion is described in the present supplementary material.

Stating the review method and the techniques to apply for the analysis before gathering a database is necessary to define which metadata need to be collected from the scientific literature search engine. For the present study, citations and references associated with each publication of the analyzed corpus must be collected to carry out the co-citation analysis and bibliographic coupling.

1.1.3 Collecting scientific literature data

The third step is to collect the data for the analysis. The scientific literature database Clarivate *Web of Science*©, Copyright Clarivate 2021 (WoS). This database covers a large number of journals in the biomedical field and has the advantage over the *PubMed*, National Center for Biotechnology Information, database to provide citations and references when exporting the dataset. The following keywords were searched in title, abstract, author's keywords and KeyWords Plus® fields:

[penumbra]
AND [imaging OR mri OR pet OR ct OR nirs OR photoacoustic OR ”oxygen metabolism”]
AND [ischem* OR ischaem* OR stroke OR cerebrovascular disease]

Additionally, preclinical studies were excluded from the corpus[1]. Review type articles were also excluded from the analysis in order to avoid the misclustering of non-related methods co-cited in reviews. The query output provided 1285 articles. The full records and cited references were exported from the WoS database on December 6th, 2021.

1.1.4 Network generation and display

The final step is to carry out the analysis. They were performed with two different software. The Bibliometrix package for R software environment was used for programming the bibliometric analysis. The VOSviewer software was used to display the networks using the proposed unified mapping and clustering method. The network visualization method was chosen for these analysis.

1.1.4.1 Co-citation method

The co-citation analysis consists in calculating the frequency with which two articles are cited together. This method can also be applied to authors and sources, in this section the focus is to uncover core publications and major evolutions in the field, therefore these alternatives will not be explored.

The network was computed on R, using the network creator provided in the Bibliometrix package with the following parameters: analysis=”co-citation” and network=”references”. The 75 most co-cited documents were included in the analysis. This threshold increased the legibility of the network and highlighted the strongest contributions.

The resulting figure was displayed using the VOSviewer software and the association strength normalization method for the unified clustering and mapping. The cluster constituents are given in figure S1.

1.1.4.2 Bibliographic coupling method

The aim of this bibliographic coupling was to map the knowledge in the field at the era of mechanical thrombectomy. Articles ranging from 2015 to today were selected, since the fundamental knowledge uncovered with the co-citation analysis has confirmed the shift of paradigm on that key-year. This filter provided a selection of 531 articles.

The network was computed on R, using the network creator provided in the Bibliometrix package with the following parameters: analysis=”coupling” and network=”references”. The coupling was applied on the full corpus, however, only 465 documents had a significant number of references in common to be coupled by.

The resulting figure S2 was displayed using the VOSviewer software and the association strength normalization method for the unified clustering and mapping, the present version displays the publication short references as opposed to the article version where cluster nominations are displayed. The cluster constituents are given in figure S3.

The nomination and analysis of the clusters was based on a selection of articles prevailing in total and local weights:

- Total weight of the paper must be equal or above the 3rd quartile of the total weights within a cluster.
- Local weight (connections within the cluster) of the paper must be equal or above the 3rd quartile of the local weights within the cluster.

[1] NOT pre-clinical NOT rat NOT mice NOT primate NOT baboons NOT cats NOT canine NOT ”animal-model”

This method of selection reflected as follow: MRI cluster: 40 articles reviewed out of 141 articles in the cluster; CTP 2019-21: 32/101; CTP 2015-19: 25/95; EVT eligibility: 22/73; Collaterals: 8/28; APT-CEST: 9/27.

1.1.4.2.1 Further investigation of O2 metabolism imaging methods in bibliographic coupling network

The exploration of the clusters to locate the O2 metabolism related articles was carried out as follow:

1. The keyword “oxygen” was searched in all fields within the corpus of literature obtained from the query ranging from [2015-2021].
2. The obtained articles were then located within the VOSviewer display of the bibliographic coupling network.
3. Elaborations from quantity, cluster affiliation and article content were then carried out to conclude on the current state of research in the field of O2 metabolism imaging.

15O-PET related publications were excluded from this analysis.

1.1.4.2.2 Limitations of the bibliographic coupling

The misclustering of articles according to their topic is frequent with this coupling method. For instance, some MRI related articles can be found in the CTP predominant clusters. A possible interpretation of this misclustering is the appreciation of one research group for a set of references. The latter represents the baseline of their scientific work and is therefore always cited in the introduction of their paper, no matter what the main topic focuses on (imaging, reperfusion therapies, time windows...). That way, one author’s publications are clustered together and the theory on which bibliographic coupling relies, is partially biased by these baseline references.

1.1.4.3 Co-word analysis

The co-word analysis consists in thematically clustering keywords. The hypothesis for the analysis is that keywords that are frequently cited together within articles have a thematic relation. This hypothesis has numerous limitations as certain words can be used in different contexts and literature corpus within one field often contain words that are too general to assign to one cluster. When taking these limits into account, a pre-processing step was added to provide a more representative analysis.

The unit for this analysis are the author’s keywords. The aim of the pre-processing step was to bring uniformity to the author’s keywords as well as eliminate words that were too global to classify. This step consisted in homogenizing synonyms, acronyms and spellings by replacing them with a single keyword and removing a list of general terms defining the field[2]. The list of keywords to delete was defined using the following criteria: (1) Contained within the 50 most occurring author’s keywords and (2) terms defining the scope of the analysis. The impact of the pre-processing step is illustrated in the second section of the present supplementary material representing the evolution of keywords trend over the years before and after pre-processing the data.

The aim for this analysis was to bring complementary information to the co-citation analysis and bibliographic coupling. It provides thematic content to elaborate on the clusters obtained with the other methods. The network was computed on R, using the network creator provided in the Bibliometrix package with the following parameters: analysis=”co-occurrences” and network=”author_keywords”. The coupling was applied on the full corpus, 482 keywords co-occurred sufficiently to be associated in clusters.

[2] ”STROKE”, ”PENUMBRA”, ”ISCHEMIC STROKE”, ”ACUTE STROKE”, ”ACUTE ISCHEMIC STROKE”, ”CEREBRAL ISCHEMIA”, ”NEUROIMAGING”, ”ISCHEMIA”, ”CEREBRAL INFARCTION”, ”IMAGING”, ”INFARCT”, ”INFARCTION”

The resulting figure S4 was displayed using the VOSviewer software and the LinLog-modularity normalization method for the unified clustering and mapping.

The obtained clusters show a high heterogeneity in terms of keywords, therefore limiting this analysis.

1.2 Impact of pre-processing keywords

Trend topics graphs were obtained on the bilioshiny graphic interface provided by bibliometrix and displayed in figure S5. The analysis was carried out on the author's keywords field and the display limited to a word minimum frequency of 5 and 2 words per year. While graph A displays 33 words, two pairs of words are synonyms of each other(”thrombectomy”-”endovascular thrombectomy” and ”thrombolysis”-”thrombolytic therapy”), one pair differs in spelling (”stroke, acute”-”acute stroke”) and one word is an acronym of the other (”mri”-”magnetic resonance imaging”). Additionally, 9 words were deleted using the criteria described in the article. Finally, one word was a synonym of a deleted keyword and therefore filtered out as well (”brain infarction”-”cerebral infarction”). The results on graph B are closer to what we can expect from the knowledge of the field and the global tendency of imaging processing science, with words such as ”machine learning” recently emerging.

2 SUPPLEMENTARY TABLES AND FIGURES

2.1 Figures

Co-citation clusters

Red Cluster	Green Cluster	Blue Cluster	Yellow Cluster	Purple Cluster
astrup j 1981	barber pa 2000	albers gw 2006	broderick jp 2013	albers gw 2018
baird ae 1997	bivard a 2013	butcher ks 2005	furlan a 1999	berkhemer oa 2015
baird ae 1998	campbell bcv 2011	davis sm 2008	hacke w 1995	campbell bcv 2015
barber pa 1998	konstas aa 2009	furlan aj 2006	hacke w 1998	goyal m 2015
baron jc 1999	kudo k 2010	hacke w 2005	hacke w 2004	goyal m 2016
beaulieu c 1999	mayer te 2000	hacke w 2009	hacke w 2008	jovin tg 2015
darby dg 1999	miteff f 2009	lansberg mg 2012	kidwell cs 2013	nogueira rg 2018
fiehler j 2002	murphy bd 2006	olivot jm 2009	langer d 2009	powers wj 2018
furlan m 1996	parsons mw 2005	straka m 2010	marler jr 1995	saver jl 2015
heiss wd 2000	parsons mw 2007	takasawa m 2008	smith ws 2005	
hossmann ka 1994	pexman jhw 2001	wu o 2003	smith ws 2008	
jones th 1981	schaefer pw 2006	zaro-weber o 2010		
kidwell cs 2000	schramm p 2004			
kidwell cs 2003	wintermark m 2002-1			
marchal g 1996	wintermark m 2002-2			
neumann-haefelin t 1999	wintermark m 2006			
ostergaard l 1996-1				
ostergaard l 1996-2				
parsons mw 2002				
rohl l 2001				
schellinger pd 2003				
schlaug g 1999				
sobesky j 2005				
sorensen ag 1996				
sorensen ag 1999				
warach s 1996				
wu o 2001				

Figure S1. Articles constituting the co-citation clusters.

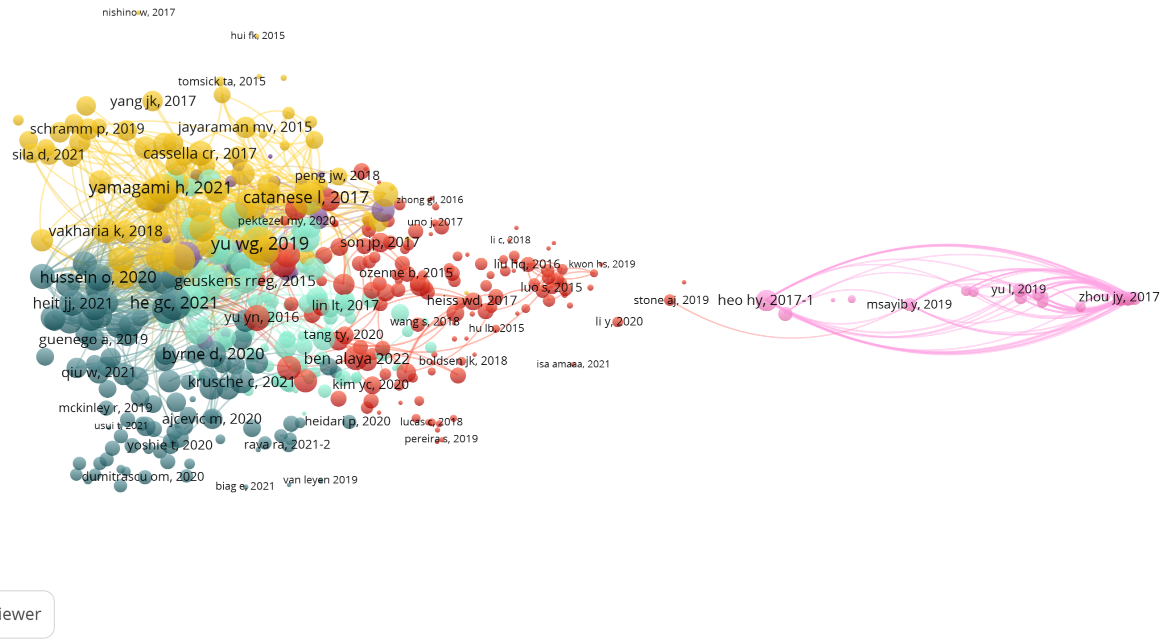

Figure S2. Bibliographic coupling at the era of mechanical thrombectomy [2015-2021] with short references to articles within the clusters. The size of the text and point is proportional to the total weight of the publication within the network.

Bibliographic-coupling clusters

**MRI Cluster**	seiler a, 2019, j cerebr blood f met	leslie-mazwi tm, 2018, neuroimag clin n am	geuskens rreg, 2015, plos one	marchese g, 2016, cor vasa
abu-samra mf, 2021, egypt j radiol nuc m	shang gf, 2020, iran j radiol	manning nw, 2018, front neurol	glen a, 2019, pol j radiol	mccabe c, 2016, j cerebr blood f met
alonso a, 2016, eur neurol	shao xf, 2020, front neurosci-switz	mckinley r, 2019, neurology	gonzalez rg, 2016, hand clinic	mctaggart ra, 2017, j neurol sci
arba f, 2019, neurol sci	shi zs, 2015, medicine	medina-rodriguez m, 2020, cerebrovasc dis	granato a, 2020, neurol sci	meiers c, 2017, am j case rep
atchaneeyasakul k, 2020, interv neurol	shi zs, 2016, j neurointerv surg	motyer r, 2018, j neurointerv surg	han yl, 2021, acta medica mediterr	menzilcioglu ms, 2015, pol j radiol
baron jc, 2020, int j stroke	simonsen cz, 2016, int j stroke	nadareishvili z, 2019, ann clin transl neur	haranhalli n, 2020, j neurosurg	mokin m, 2017, stroke
ben alaya i, 2022, clin imag	son jp, 2017, j stroke	nannoni s, 2019, neuroradiology	heit jj, 2019, radiol clin n am	na dg, 2015, korean j radiol
benzakoun j, 2021, j cerebr blood f met	song sb, 2017, plos one	olivot jm, 2021, stroke	hoelter p, 2020, neuroradiology	nael k, 2016, magn reson imaging c
bhattacharjee r, 2021, nmr biomed	steiner l, 2021, sci rep-uk	oostema ja, 2020, curr emerg hosp me r	hong l, 2019, ann neurol	nishino w, 2017, j stroke cerebrovasc
boldsen jk, 2018, front neuroinform	stier n, 2015, ieee int c bioinform	ospel jm, 2021, j stroke	horsch ad, 2016, clin neuroradiol	ozdemir ao, 2020, j clin neurosci
brodtmann a, 2015, front hum neurosci	stone aj, 2019, hum brain mapp	ostman c, 2020, front neurol	huang l, 2017, world neurosurg	peng jw, 2018, exp ther med
campbell bcv, 2015, int j stroke	su h, 2020, int j clin exp patho	peerlings d, 2021, eur radiol	huang xy, 2015, lancet neurol	petersen nh, 2019, stroke
cao j, 2017, transl med res	takahashi s, 2015, j stroke cerebrovasc	perez-pelegri m, 2021, j x-ray sci technol	huang xy, 2017, j neuroimaging	phan k, 2019, j neurointerv surg
chen cy, 2015, plos one	tang ty, 2018, ebiomedicine	petrov i, 2021, neurosonology cereb	kamran m, 2015, am j neuroradiol	protto s, 2016, cardiovasc inter rad
cho th, 2015, stroke	tang ty, 2020, j neurol	pozzi-mucelli ra, 2021, neurologist	karaalioglu b, 2018, j comput assist tomo	pruvost-robieux e, 2018, front neurol
chung jw, 2017, j stroke cerebrovasc	tang zw, 2015, j neurochem	psychogios mn, 2021, clin neuroradiol	karwacki gm, 2017, j comput assist tomo	psychogios mn, 2017, neuroradiol j
clerigues a, 2020, comput meth prog bio	uno j, 2017, cerebrovasc dis	qiu w, 2021, j stroke	kasasbeh as, 2019, stroke	puig j, 2020, j neuroimaging
cuciureanu di, 2019, rev chim-bucharest	uwano i, 2017, magn reson med sci	rava ra, 2020, am j neuroradiol	kate m, 2018, j stroke	pushie mj, 2018, acs chem neurosci
cui ys, 2019, int j gerontol	voglis s, 2021, sci rep-uk	rava ra, 2020, neuroradiol j	kate m, 2019, j cerebr blood f met	rotem sh, 2020, j neuroimaging
dani ka, 2017, j cerebr blood f met	vupputuri a, 2017, p ann int ieee embs	rava ra, 2021, j neurointerv surg	kate m, 2021, can j neurol sci	sallustio f, 2017, j neurointerv surg
darwish eaf, 2020, insights imaging	vupputuri a, 2021, comput biol med	rava ra, 2021, neuroradiol j	kawano h, 2017, brain	schramm p, 2019, j neurointerv surg
de la rosa e, 2021, med image anal	vupputuri a, 2021, j neurosci meth	renu a, 2019, j neurointerv surg	kawiorski mm, 2016, j stroke cerebrovasc	sila d, 2021, brain sci
debatisse j, 2019, j cerebr blood f met	wang tl, 2017, biomed res-india	rotkopf lt, 2020, j neuroimaging	liao ch, 2020, j chin med assoc	smith ws, 2019, neurotherapeutics
debs n, 2020, comput biol med	wang tl, 2017, technol health care	sarraj a, 2021, stroke	lin cj, 2016, medicine	spiotta am, 2015, j neurointerv surg
dehkharghani s, 2015, am j neuroradiol	wang yr, 2021, neuropsych dis treat	scheldeman l, 2021, stroke	lin lt, 2016, radiology	sussman es, 2016, world neurosurg
dehkharghani s, 2017, am j neuroradiol	watson cg, 2016, stroke	seners p, 2021, ann neurol	lin lt, 2017, j cerebr blood f met	tomsick ta, 2015, j neurointerv surg
dejobert m, 2016, j stroke cerebrovasc	werner p, 2015, j cerebr blood f met	shahedah kk, 2018, j roy coll phys edin	lin lt, 2018, stroke	turk as, 2019, lancet
efrati s, 2018, front psychol	werner p, 2016, neurology	shi fn, 2019, radiology	liu n, 2017, mol neurobiol	vakharia k, 2018, neurosurg clin n am
fang rg, 2015, comput med imag grap	wright ea, 2016, plos one	shi z, 2021, front neurosci-switz	luby m, 2016, int j stroke	warach sj, 2016, stroke
gersing as, 2015, neuroradiology	wu hm, 2021, plos one	siegler je, 2020, clin neurol neurosur	luo yj, 2020, j craniofac surg	wu l, 2018, neuroimag clin n am
giacalone m, 2017, magn reson med	wu xj, 2017, medicine	siegler je, 2020, j neuroimaging	man fy, 2015, neuroradiology	yamagami h, 2021, neurol med-chir
gupta a, 2019, ieee eng med bio	xu kx, 2020, medicine	siegler je, 2020, neurology	man y, 2019, acta medica mediterr	yang jk, 2017, biomed microdevices
heiss wd, 2017, j nucl med	xu zh, 2019, front neurol	simon m, 2020, pro biomed opt imag	manniesing r, 2016, peerj	yu wg, 2019, front neurol
hiramatsu r, 2016, bmc neurol	yamaguchi s, 2018, plos one	sotoudeh h, 2019, acad radiol	mikkelsen ik, 2015, eur radiol	zhang wy, 2021, int j gen med
hsia aw, 2019, stroke	yang lj, 2018, medicine	sotoudeh h, 2019, emerg radiol	moghari md, 2021, phys med biol	**Collateralization Cluster**
hu lb, 2015, chinese med j-peking	yu y, 2019, j cerebr blood f met	stella ab, 2021, clin physiol funct i	moreau f, 2016, neurophotonics	agarwal s, 2018, neurology
huang kl, 2017, curr neurovasc res	yu y, 2021, am j neuroradiol	sui bb, 2020, j int med res	morris rs, 2018, brain	caruso p, 2019, neurologist
huang kl, 2020, j neuroinflamm	yu yn, 2020, jama netw open	suomalainen op, 2021, j neurol sci	mosqueira aj, 2020, neurologia	conrad j, 2020, j neurol
hur w, 2018, medicine	yuan t, 2018, j magn reson imaging	suzuki k, 2021, heart vessels	murayama k, 2017, j comput assist tomo	ernst m, 2015, am j neuroradiol
irvine hj, 2018, j cerebr blood f met	zaro-weber o, 2017, j cerebr blood f met	tortuyaux r, 2020, rev neurol-france	najm m, 2018, can j neurol sci	heit jj, 2018, neuroimag clin n am
isa amaaa, 2021, pertanika j sci tech	zaro-weber o, 2019, ann neurol	usui t, 2021, j med case rep	padroni m, 2016, plos one	higazi mm, 2016, egypt j radiol nuc m
jiang l, 2021, quant imag med surg	zhang s, 2015, cns neurosci ther	vagal a, 2018, stroke	pan jw, 2017, neural regen res	jiang b, 2019, int j stroke
kim yc, 2020, j clin med	zhang s, 2017, int j stroke	van der meij a, 2020, trials	pennig l, 2020, sci rep-uk	kawano h, 2016, stroke
klug j, 2021, j cerebr blood f met	zhang sx, 2015, j huazhong u sci-med	van leyen k, 2019, j cerebr blood f met	psychogios k, 2019, neurologist	kubiak-balcerewicz k, 2017, j stroke cerebrovasc
kohno n, 2016, j stroke cerebrovasc	zhang x, 2017, am j neuroradiol	van ommen f, 2019, med phys	reeves p, 2018, plos one	landowski lm, 2020, semin thromb hemost
kuribara t, 2015, neurol med-chir	zhong gl, 2016, transl stroke res	vanicek j, 2019, j stroke cerebrovasc	slotty pj, 2015, j neurosurg	li cc, 2017, neurol asia
kwon hs, 2019, j neurol	zhou y, 2017, eur radiol	visser mj, 2021, stroke	songsaeng d, 2020, acta radiol	li cc, 2018, neural regen res
lee gh, 2015, j neuroimaging	**CTP [2019-2021] Cluster**	xia q, 2019, clin radiol	sotoudeh h, 2019, iran j radiol	lin lt, 2021, neurology
lee h, 2020, j digit imaging	ajcevic m, 2020, physiol meas	yaghi s, 2019, j neuroimaging	ukmar m, 2017, brit j radiol	liu c, 2018, eur radiol
lee kj, 2017, j stroke cerebrovasc	berndt mt, 2020, am j neuroradiol	yaghi s, 2021, stroke	van os hja, 2016, neurology	luo cm, 2017, brain pathol
lee sh, 2017, j clin neurol	berndt mt, 2021, eur radiol	yoshie t, 2020, am j neuroradiol	wang s, 2018, biomed opt express	lutz y, 2020, math biosci eng
legrand l, 2015, am j neuroradiol	beutler bd, 2021, am j case rep	zhang r, 2020, ann palliat med	wannamaker r, 2018, stroke	mukherjee a, 2017, j stroke cerebrovasc
li c, 2018, restor neurol neuros	biag e, 2021, neurologist	zhang s, 2020, front neurosci-switz	wood rp, 2015, proc spie	nguyen th, 2020, case rep neurol
li y, 2020, brain	bogh n, 2021, j cerebr blood f met	zhu gm, 2020, int j stroke	wu b, 2018, front neurol	oh sh, 2018, ther hypothermia tem
lin ch, 2016, curr neurovasc res	broocks g, 2021, j neurol	**CTP [2015-2019] Cluster**	yu yn, 2016, sci rep-uk	potreck a, 2017, eur radiol
liu hq, 2016, int j clin exp med	brugnara g, 2020, stroke	abdelgawad ea, 2017, j neuroradiology	**MT Eligibility Cluster**	rusanen h, 2015, cerebrovasc dis
liu ll, 2019, neurocomputing	byrne d, 2020, can assoc radiol j	agarwal s, 2015, front neurol	aydin e, 2016, med j bakirkoy	rusanen h, 2015, cerebrovasc dis-a
liu pb, 2019, lect notes comput sc	campbell bcv, 2019, lancet neurol	ajcevic m, 2021, ann biomed eng	boers amm, 2017, j cerebr blood f met	tahir ra, 2021, neuroradiology
livne m, 2017, stroke	cao w, 2020, am j neuroradiol	alemseged f, 2019, int j stroke	bonnard t, 2019, stroke	varadharajan s, 2016, am j emerg med
lopez-mejia m, 2016, j stroke cerebrovasc	carbone f, 2019, transl stroke res	amidon rf, 2021, cureus	bonney pa, 2019, neurosurgery	von baumgarten l, 2016, neuroradiology
lorenzano s, 2019, neurology	chen cs, 2021, front neurol	austein f, 2018, eur radiol	borst j, 2015, stroke	weinberg jh, 2020, interdiscip neurosur
lu xd, 2021, eur radiol	chen wh, 2019, world neurosurg	ayekpam m, 2015, j evol med dent sci-	bouchez l, 2017, eur j radiol	wintermark m, 2015, int j stroke
lucas c, 2018, front neurol	christensen s, 2021, j cerebr blood f met	barlinn k, 2015, clin neuroradiol	carrera e, 2017, neurology	xu sm, 2019, exp ther med
luo s, 2015, j neuroradiology	chung jw, 2019, j stroke	bennink e, 2015, plos one	caruso p, 2018, neurol sci	**APT CEST Cluster**
luo s, 2018, neurol sci	chung kj, 2021, pro biomed opt imag	benson j, 2015, eur j radiol	cassella cr, 2017, emerg med clin n am	foo ls, 2020, j magn reson
luo y, 2017, eur radiol	cimflova p, 2020, j stroke cerebrovasc	bill o, 2019, am j neuroradiol	catanese l, 2017, circ res	hampton dg, 2021, semin ultrasound ct
ma h, 2015, int j stroke	de havenon a, 2021, j neurosurg	bivard a, 2015, brain	chen kn, 2017, stroke vasc neurol	harston g, 2016, j cerebr blood f met
mckinley r, 2017, j cerebr blood f met	de vis jb, 2019, eur radiol	bivard a, 2016, ann neurol	comai a, 2015, radiol med	harston gwj, 2015, brain
mckinley r, 2018, front neurol	desai sm, 2020, j neurointerv surg	bivard a, 2016, neuroradiology	devlin tg, 2015, ann vasc surg	heo hy, 2016, magn reson med
modrau b, 2016, eur stroke j	desai sm, 2021, neurology	bivard a, 2017, ann neurol	el-tawil s, 2017, int j stroke	heo hy, 2017, magn reson med
molad ja, 2017, j stroke cerebrovasc	di iorio r, 2021, cerebrovasc dis extr	bivard a, 2017, circulation	gawlitza m, 2016, j stroke cerebrovasc	heo hy, 2017, magn reson med-a
morelli n, 2015, intern emerg med	dumitrascu om, 2020, j neuro-ophthalmol	bivard a, 2017, neurology	goktay ay, 2017, adv exp med biol	huang sm, 2015, sci rep-uk
motta m, 2015, front neurol	fan ap, 2020, j cerebr blood f met	bivard a, 2017, stroke	gur-ozmen s, 2019, medicine	jiang wp, 2016, contrast media mol i
muhammad a, 2019, bmj case rep	fan sy, 2019, front neuroinform	bivard a, 2018, front neurol	hasegawa h, 2015, j neurosurg	jones km, 2018, j magn reson imaging
mulder ia, 2019, int j stroke	federau c, 2019, neurology	borst j, 2015, plos one	hillis ae, 2015, front neurol	khlebnikov v, 2018, magn reson med
mundiyanapurath s, 2016, eur j radiol	furlanis g, 2020, j neurol	breuer l, 2015, j stroke cerebrovasc	hiramatsu r, 2018, interv neuroradiol	kim h, 2022, magn reson med
mundiyanapurath s, 2017, eur j radiol	garcia-esperon c, 2020, cns neurosci ther	cereda cw, 2016, j cerebr blood f met	hosseini mb, 2018, j neuroimaging	li cm, 2017, front neurosci-switz
nguyen hbt, 2020, case rep neurol	garcia-esperon c, 2021, stroke	chen cs, 2017, stroke	hou qh, 2017, int j stroke	li sh, 2020, front aging neurosci
noguchi k, 2017, j stroke cerebrovasc	guenego a, 2019, stroke	chen cs, 2019, j cerebr blood f met	hui fk, 2015, j neurointerv surg	ma xy, 2017, sci rep-uk
olivot jm, 2017, rev neurol-france	han qd, 2020, medicine	cheripelli bk, 2016, int j stroke	inoue a, 2016, int j surg case rep	momosaka d, 2020, plos one
ozenne b, 2015, j neuroimaging	hassan ae, 2020, interv neurol	cheripelli bk, 2016, stroke	jang hg, 2019, j korean neurosurg s	msayib y, 2019, neuroimage-clin
park mg, 2017, j neuroradiology	he gc, 2021, rev neuroscience	chidambaram pk, 2015, j evol med dent sci-	jayaraman mv, 2015, j neurointerv surg	promjunyakul no, 2016, j cerebr blood f met
payabvash s, 2017, acta radiol	heidari p, 2020, front neurol	de havenon a, 2017, stroke vasc neurol	jiang b, 2017, stroke	sun p, 2019, j cerebr blood f met
pektezel my, 2020, j stroke cerebrovasc	heit jj, 2021, j neuroimaging	de souza a, 2021, neurol asia	jung s, 2021, j stroke cerebrovasc	sun pz, 2022, magn reson med
pereira s, 2018, med image anal	hoelter p, 2019, neuroradiology	d'esterre cd, 2015, neurol sci	kaiser d, 2017, aktuel neurol	wang zx, 2020, j neurosci meth
pereira s, 2019, ieee t med imaging	hussein o, 2020, egypt j neurol psych	d'esterre cd, 2015, stroke	kameda k, 2018, j neurointerv surg	worthoff wa, 2019, new dev nmr
peretz s, 2018, j neurointerv surg	jadhav ap, 2020, neurol clin	d'esterre cd, 2015, transl stroke res	kass-hout t, 2015, j neurointerv surg	yu l, 2019, front neurol
piliszek a, 2016, neurol neurochir pol	jeong hg, 2019, stroke	dharmasaroja pa, 2015, j neurosci rural pra	kemmling a, 2015, j cerebr blood f met	zhang xy, 2017, nmr biomed
polan rm, 2015, am j neuroradiol	karakus a, 2021, bmj open	doucet c, 2016, j comput assist tomo	kidwell cs, 2015, neurol clin	zhang y, 2017, magn reson med
qiu yg, 2021, front aging neurosci	karwacki gm, 2019, j neuroradiology	eckert b, 2015, aktuel neurol	kim ey, 2016, eur radiol	zhao y, 2019, magn reson med
reimer j, 2018, cerebrovasc dis	kim bj, 2018, cerebrovasc dis	el-tawil s, 2019, stroke	kleine jf, 2017, stroke	zhou jy, 2017, cest imaging: advances and applications
rudilosso s, 2019, am j neuroradiol	kim bj, 2021, jama neurol	flottmann f, 2017, sci rep-uk	koh e, 2017, j stroke cerebrovasc	
sathish r, 2019, ieee eng med bio	kim jm, 2020, neurol sci	fotakopoulos g, 2018, futur sci oa	lansberg mg, 2017, ann neurol	
savic t, 2019, p natl acad sci usa	kim yc, 2019, proc spie	fotakopoulos g, 2018, j stroke cerebrovasc	leiva-salinas c, 2016, stroke	
scalzo f, 2015, front neurol	krusche c, 2021, clin hemorheol micro	fu jj, 2020, transl stroke res	longo m, 2018, radiol med	

Figure S3. Articles constituting the bibliographic coupling clusters.

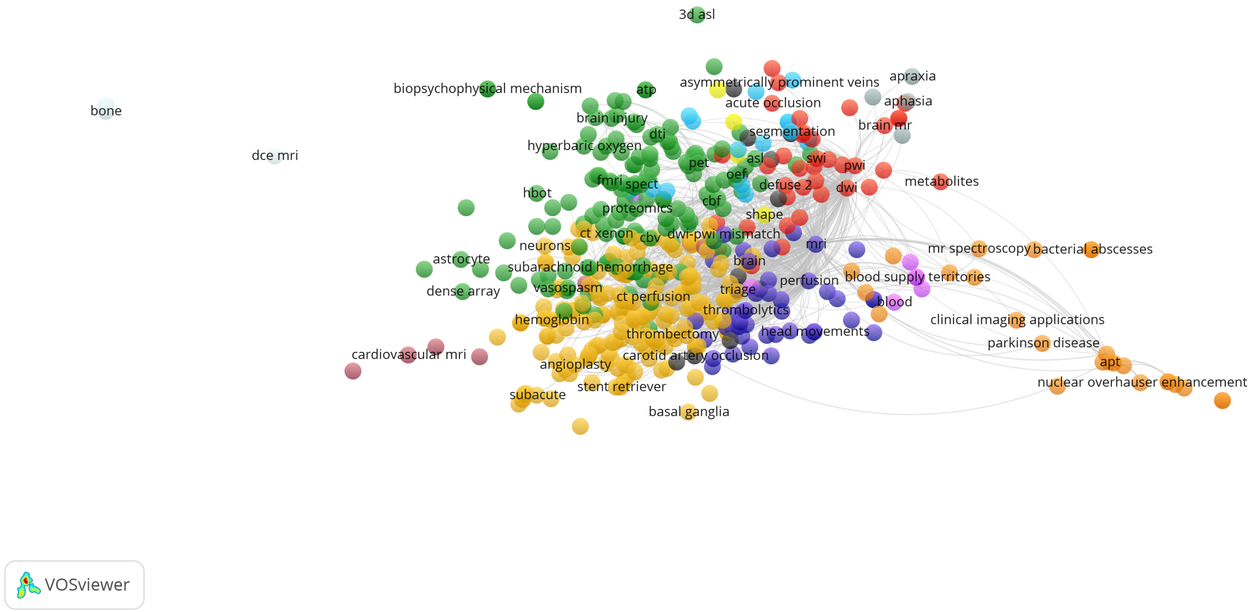

Figure S4. Co-word analysis network normalized with LinLog/modularity VOSviewer method.

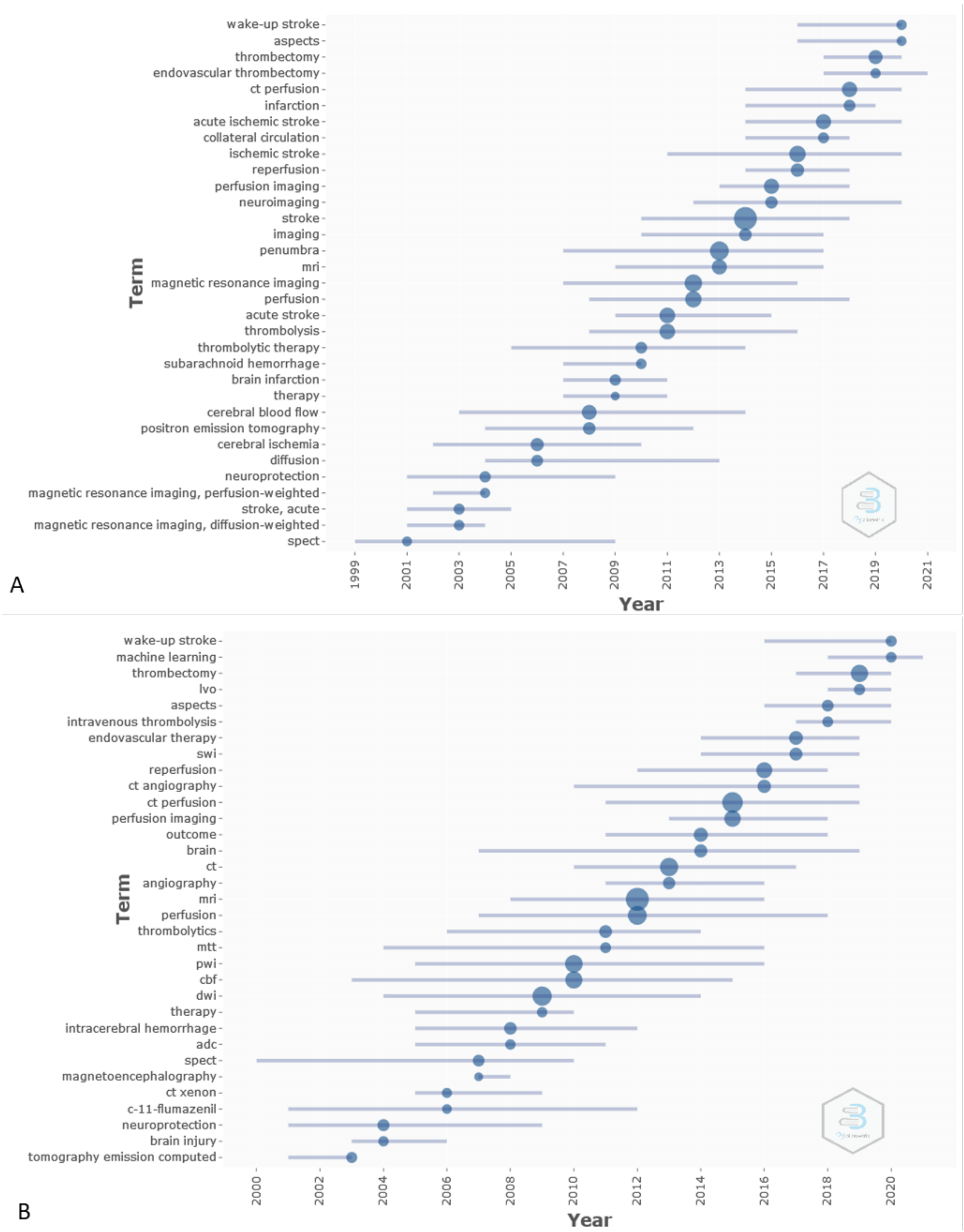

Figure S5. Trend topics analysis based on author's keywords before **(A)** and after **(B)** pre-processing.
